# Mild chronic stress promotes female fertility via the ovarian CRF receptor

**DOI:** 10.1186/s12964-025-02371-0

**Published:** 2025-08-14

**Authors:** Eran Gershon, Orna Issler, Mariana Schroeder, Yael Kuperman, Nava Nevo, Shlomi Lazar, Michal Elbaz, Nava Dekel, Alon Chen

**Affiliations:** 1 Department of Ruminant Science, Institute of animal science, Agricultural Research organization, The Volacani Center, Rishon LeZion, 750510 Israel; 2 Department of Veterinary Resources, the Weizmann Institute of Science, Rehovot, 76100 Israel; 3 Department of Immunology and Regenerative Biology, the Weizmann Institute of Science, Rehovot, 76100 Israel; 4 Department of Pharmacology, the Israeli Institute for Biological Research (IIBR), Ness- Ziona, Israel; 5 Present address, NYU Grossman School of Medicine, NYU, New York, NY USA; 6 Present address, Institute of Biochemistry and Molecular Medicine, Bern University, Bern, Germany; 7 Department of Molecular Neuroscience, Weizmann Institute of Science, Rehovot, 76100 Israel

**Keywords:** Chronic variable stress, Corticotrophin releasing factor receptor, Ovary, Reproduction, Ovulation, Baby boom, Estrogen

## Abstract

**Background:**

In many species, including human, stress is accompanied by disruption of reproductive functions. The endocrine stress-response is activated and regulated by members of the corticotropin releasing factor (CRF) protein family. Stress stimuli may affect reproductive functions locally, recruiting autocrine/paracrine strategies. Yet, the molecular mechanisms mediating these effects are not fully understood.

**Methods:**

To explore the molecular mechanism mediating the ovarian stress response, we used three different models: (1) ICR mice subjected to chronic variable stress (CVS) procedure for 4 weeks. The stress procedure consisted of 9 different stressors per week, approximately 2 stressors per day both in the dark and the light phases. (2) wild-type mice undergoing intraovarian injection of the CRF receptor antagonist, β-asstressin, and (3) CRF-R1 knockout mice.

**Results:**

We report herein that ovulation rate was significantly elevated, and the litter size was substantially increased, in the following estrous cycle of female mice subjected to mild chronic variable stress (CVS). These females exhibited lower serum estrogen levels associated with reduced ovarian 17β-HSD3 expression. Exploration of the involvement of a neuroregulatory mechanism in this event revealed upregulation of the corticotropin releasing factor type 1 receptor (CRFR1) in the theca-interstitial cells of large ovarian follicles. In agreement, CRFR1 knockout mice, as well as wild-type females undergoing intraovarian injection of the CRF receptor antagonist, β-asstressin, displayed reduced ovulation rate, enhanced estrogen secretion and an increase in 17β-HSD3 expression.

**Conclusions:**

Our findings show a direct gonadal response to neuroendocrine and central stress-response regulators. The mechanism of this unexpected beneficial effect of CVS on reproduction may provide a neuro-endocrine background to the well-known “Baby Boom” phenomenon.

**Supplementary Information:**

The online version contains supplementary material available at 10.1186/s12964-025-02371-0.

## Background

Adaptation to challenges involves a few coordinated autonomic, metabolic, neuroendocrine and behavioral changes known collectively as the stress response. Over the last half of the century, an extraordinary range of stress mediated effects have been uncovered demonstrating profound effects on many of the organism’s regulatory systems. Although the effects of stress on homeostasis through regulation of the immune system, metabolic functions, neuronal and behavior outputs are well known and have been reviewed extensively [1, 2] its role in regulating reproductive functions received considerably less attention [1, 2].

Stress has been shown to disrupt reproductive functions in many species, including human [3]. Specifically, severe stress may lead to the suppression of the menstrual cycle, an effect referred to as functional hypothalamic chronic anovulation characterized by ovarian quiescence, amenorrhea and infertility [4, 5]. Moreover, this clinical syndrome has been associated with various disturbed life style variables, such as eating disorders and excessive exercising, with the subsequent extreme weight loss [6–9].

The endocrine stress-response is activated and regulated by the brain peptide, corticotropin releasing factor (CRF [10]). The effects of CRF-related peptides are mediated by two known receptors, CRF receptor type 1 and type 2 (CRFR1 and 2 respectively [11–13]). Interestingly, the expression of CRF and CRFR1 is not limited to the brain but rather demonstrated in peripheral organs including the reproductive tissues, such as the ovary, testis, placenta and endometrium [14–17]. The fact that, in addition to their expression in the hypothalamic brain nuclei, the CRF peptide family members and their corresponding receptors exist in reproductive organs, may suggest that along with the classical systemic manner [18] stress stimuli may affect reproductive functions locally, employing autocrine/paracrine strategies. Yet, the molecular mechanisms mediating these effects are not fully understood.

## Methods

### In vivo protocols

#### Animals

For CVS procedure, we used 8-week-old ICR female mice (Envigo, Rehovot, Israel). ICR (also known as CD-1) outbred mice, originally derived from Swiss albino mice, are widely used in biomedical research due to their high reproductive performance. The females were housed for acclimation as described below for 2 weeks prior to CVS procedure. For all other experiments we used C57BL/6 females (Envigo, Rehovot, Israel). We also employed females lacking the expression of CRFR1 (CRFR1 knock out (KO) females) and CRFR1-GFP reporter transgene mice described previously [19, 20]. Mice were housed and handled in a pathogen-free, temperature-controlled (22 °C ± 1 °C) on a 12/12 h light/dark cycle, with lights switched on at 6 a.m. Animals were fed a regular chow diet (2018 Teklad Global 18% Protein Rodent Diet). Animals were given ad libitum access to food and water. Food was withdrawn only if required for an experiment. Mice testing, estrous cycle monitoring and tissues collection were performed at morning hours (between 7:30 to 10:00 a.m.). The Institutional Animal Care and Use Committee of The Weizmann Institute of Science approved all procedures.

#### Superovulation protocol

Sexually immature, 24-day-old, either C57BL/6 wild type (WT) or CRF-R1 KO female mice, were injected with 5 IU of pregnant mare serum gonadotropin (PMSG; Chrono-gest Intervest, Oss, The Netherlands), followed, 48 h later, by 5 IU human chorionic gonadotropin (hCG, Chrono-gest Intervest). Twenty hours later the oocytes present in the oviduct were counted to examine ovulation. Ovaries isolated at various time points, either before or after administration of hCG, were subjected to the analysis described below.

The specific stages of the estrous cycle in sexually mature, cycling female ICR mice (7–9 wk old) were monitored by microscopic analysis of the vaginal cell population. At the indicated days, females were taken for further analysis.

#### CRF localization in the ovary

Ovaries from mice expressing GFP extensively colocalized with CRF-R1 [19] were fixed in 4% paraformaldehyde in borate buffer, pH 9.5 followed by immersion in 30% sucrose in the same fixative at 4˚C. The ovaries were then frozen and sectioned at 25µM using a sliding microtome and stored in PBS at 4˚C. Sections were blocked in 3% fetal calf serum in TBST for 30 min. The sections were then incubated with anti-GFP antibody (Sigma-Aldrich, Rehovot, Israel 1:500) overnight at 4 °C. Sections were washed with TBST and immonureacted with the Alexa 488-conjugated secondary antibody for 1 h at room temperature, washed again with TBST. Sections were visualized using a fluorescent microscope (E600, Nikon, Tokyo Japan).

#### CVS procedure

The stress regime was modified from Willner works with mice [27] and rats [28]. Eight-week-old ICR mice were subjected to the CVS procedure for 4 weeks. The stress procedure consisted of 9 different stressors per week, approximately 2 stressors per day both in the dark and the light phases. The stressors were scheduled in 2 different weekly timetables, which were repeated twice to create unpredictability. The weekly stressors regime included: unpredictable illuminations during the dark phase, 36 h of continuous lighting, two periods of housing in a male cage for 4 h at a time, 2 periods of 30^0^ cage tilt for 6–18 h, damp bedding with 200 ml water for 17 h, 2 periods low-intensity stroboscopic illumination in 180 strobes/min for 5 h each, two periods (10 min each) of 80 dB white noise for 5 h each, two periods of restraint stress within a ventilated 50 ml tube, 4 h of either food or water restriction, a 4-hour-stay in a cage with no bedding, followed by 20 min of water within that cage. Females were weighed once a week during the CVS procedure. At the end of the CVS procedure, mice were not stressed for 48 h prior to starting the different tests, to ensure any effects of the chronic stress were not due to the last stressor.

#### Treatment with β-asstressin

Ovarian intrabursal injection was performed in lightly anesthetized sexually immature, 25-day-old, PMSG-primed C57/bl mice. For this purpose their ovaries were pulled out through a small lumbosacral incision and phosphate-buffered saline (100 µl) with or without β-asstressin (500 µM) was injected through a 30-gauge needle threaded into the ovarian bursa. After injection, the ovary was returned to the abdominal cavity, and the skin was clipped. The animals were taken for further analysis as described in the text. For ovulation examination, this procedure was followed by an hCG (10 IU) intraperitoneal injection. Twenty-two hours later, the mice were sacrificed by cervical dislocation, the ampullae of the oviducts were excised, and the oocytes released and counted under the microscope. For estrogen determination, animals were sacrificed 3 h after β-asstressin injection. Estrogen levels were determined as described here. 

#### RNA preparation and real-time PCR analysis

Total RNA was isolated from the ovaries of hormonally treated immature 25-day-old WT C57BL/6 female mice treated with or without β-asstressin or CRF-R1 KO females undergoing the ovulation induction protocol described above, as well as from naïve and CVS-exposed females. Ovaries were immediately placed on dry ice and stored at −80˚C. RNA was extracted using a 5 PRIME Manual PerfectPure RNA Cell & Tissue kit (5 Prime GmbH, Hamburg, Germany). Extracted RNA was treated with RQ1 DNAse (Promega, Madison, WI, USA) to avoid false-positive results due to DNA contamination. The concentration and purity of the RNA were measured and determined using nanodrop (Thermo scientific, Airport city, Israel). Only high purity RNA was taken for analysis. The RNA samples were reverse transcribed to generate cDNA pools that were later used as templates for quantitative real-time PCR analysis using specific primers. The expression of HPRT mRNA served as the internal control. The real-time reaction was performed in an AB7500 thermocycler using fluorescent SYBR Green technology (ABgene, Epsom, Surrey, UK). The following specific primers were designed using Primer Express software (PE Applied Biosystems, Perkin Elmer, Foster City, CA, USA), all primers were designed on exon-exon junctions or two different exons: mCRFR1 primers: 5’TGCCAGGAGATTCTCAACGAA3’ and 5’AAAGCCGAGATGAGGTTCCAG3’ corresponding to nucleotides 495–515 and 656–676 respectively. CRF primers: 5’GCAGTTAGCTCAGCAAGCTCAC3’ and 5’CAAATGATATCGGAGCTGCG3’ corresponding to nucleotides 683–705 and 824–844 respectively. 17ß-HSD type 3 primers: 5’AGGTTCTCGCAGCACCTTTTT3’ and 5’CATCGCCTGCTCCGGTAAT3’ corresponding to nucleotides 137–157 and 218–236 respectively. Aromatase primers: 5’GTCGAAGCAGCAATCCTGAAG3’ and 5’CTGGTACCGCATGCTTTCATT3’ corresponding to nucleotides 1028–1048 and 1127–1147 respectively. HPRT primers: 5’GCAGTACAGCCCCAAAATGG3’ and 5’GGTCCTTTTCACCAGCAAGCT3’ corresponding to nucleotides 540–559 and 571–591 respectively. The PCR conditions were as follows: cDNA equivalent to 1 µg of total RNA was amplified using PCR for 45 cycles at an annealing temperature of 60 °C. Each qPCR reaction contained 10 µl 2 x SYBR Green Mastermix, a final primer concentration of 250 nM, and 10 ng of template cDNA. The specificity of the amplification products was checked by melting curve analysis (supplementary Figure 1). Products from the real time PCR were sent to sequencing analysis with the relevant specific primers to validate the correct sequences of the genes were amplified. Relative expression levels (*ΔΔ*Ct) were calculated by normalizing to hypoxanthine guanine phosphoribosyl transferase (HPRT).

#### RNase protection assay

Total RNA was extracted from different WT C57BL/6 mouse tissues using the TRI Reagent RNA isolation reagent (Molecular Research Center, Cincinnati, OH). CRF-R1 and glyceraldehyde-3-phosphate dehydrogenase (GAPDH; Anbion Inc., Austin, TX) mRNA levels were measured simultaneously by RNase protection, using mouse GAPDH as the internal loading control. A 6CRF-R1 antisense riboprobe specific to the mCRF-R1 mRNA was synthesized by using T3 RNA polymerase. An antisense riboprobe specific to mouse GAPDH mRNA was synthesized by using T3 RNA polymerase. All riboprobes were synthesized in the presence of [α-^32^P]uridine 5′-triphosphate (UTP) [3000 Ci/mmol (1 Ci = 37 GBq)] and either 20 µM UTP for CRF-R1 or 200 µM UTP for GAPDH. The fragment sizes protected by CRF-R1 and GAPDH riboprobes are 592 and 316 nucleotides, respectively.

RNA samples (40 µg of peripheral tissues or 20 µg of brainstem/cerebellum tissues) were hybridized in 24 µl of deionized formamide plus 6 µl of hybridization buffer containing 6 × 10^5^ cpm of CRF-R1 and 4 × 10^4^ cpm GAPDH antisense riboprobes. After heating to 85 C for 5 min, the samples were hybridized at 42 C for 15 h and subsequently digested by RNase (100 µg/ml RNase A and 0.5 U/ml RNase T1) at 24 C for 60 min. The samples were resolved on 4% polyacrylamide 7 M urea gels. Image analysis was performed by using the PhosphorImager system (Molecular Dynamics, Inc., Jersey City, NJ) and the ImageQuant TL 4.0 software package (Amersham Biosciences, Piscataway, NJ).

#### Determination of serum estrogen

Estrogen was extracted from serum of hormonally treated immature 25-day-old WT C57BL/6 female mice treated with or without β-asstressin or CRF-R1 KO females as well as of naïve and CVS-exposed females on proestrous day using petroleum ether, which was subsequently evaporated. The extracts were reconstituted with assay buffer. Estrogen serum levels were determined using EIA kit according to the manufacturer instructions (Cayman Chemical, Ann Arbor, MI, USA).

#### Human birth rate data

All data of human birth rate was retrieved from the Israel Central Bureau of Statistics data base upon our request. The data was analyzed with their assistance.

#### Statistical analyses

Differences between 2 the groups examined were determined busing the unpaired Student’s *t* test with a confidence level of 95% (JMP IN Statistical Discovery Software, version 7.0, SAS Institute Inc., Cary, NC, USA). All estimates of central tendency are mean values with standard errors of the mean (SEM).

## Results

We used the well-documented mild chronic variable stress (CVS) model to unravel the direct role of CRF/CRFR in the ovary. In this model, mice are exposed to a daily, long-term, non-predictable mild stressor as described in details under Materials and Methods. Our and others previuos studies showed that mice exposed to this CVS regimen exhibit an increased depression-like behavior particularly anhedonia, reduced weight gain and hair qulity as well as increased adrenal size [21–23] As expected, sexually mature ICR female mice, undergoing CVS for 4 weeks in the present sdudy, gained less weight than control animals (supplementary Figure 2).

**Fig. 1:**
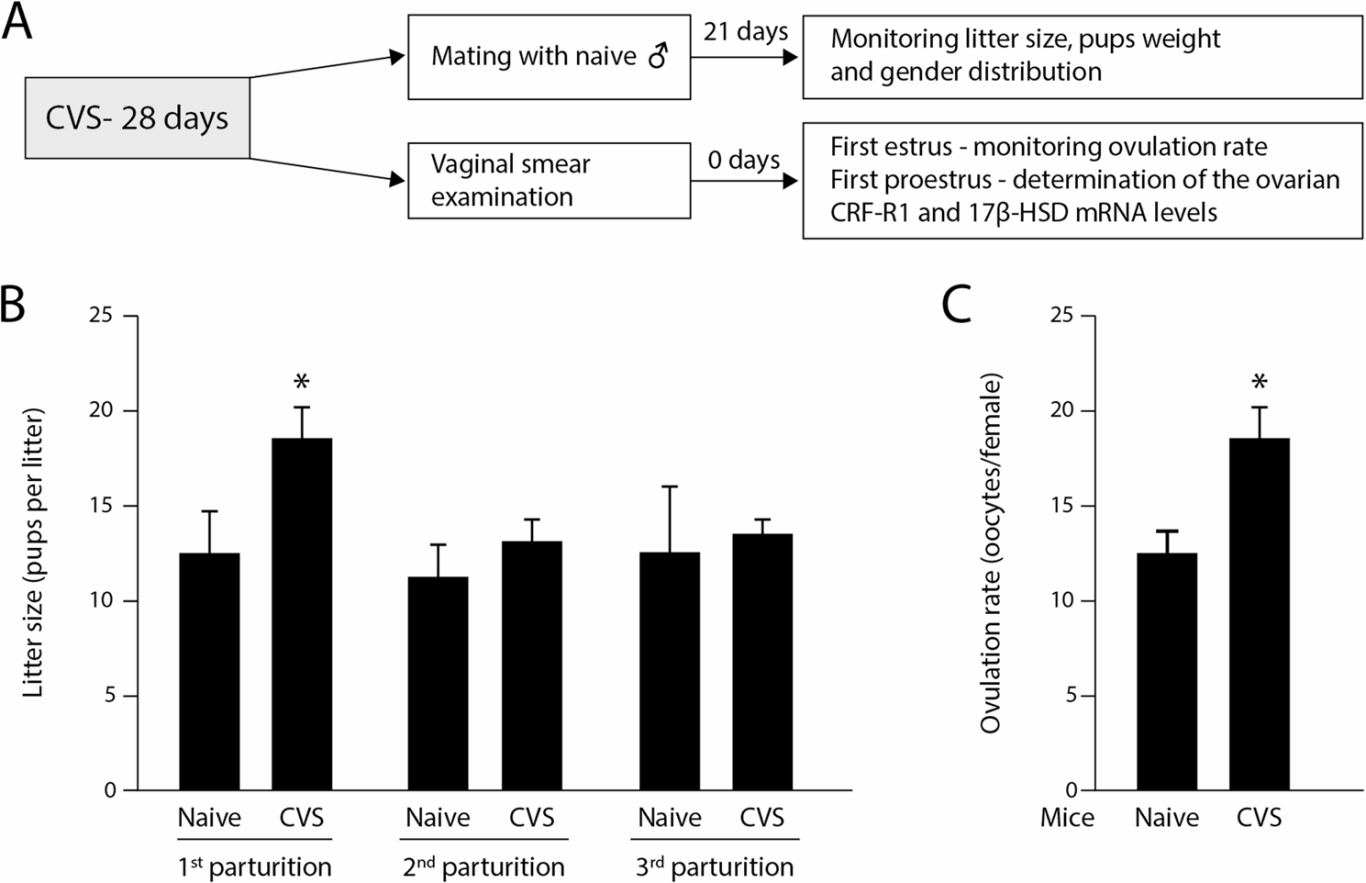
Ovulation rate and litter size are higher in CVS females. **A** A schematic presentation of the experimental protocol. A total of 3 CVS experiments was performed as described under Materials and Methods. **B** Females that did not undergo CVS (Naïve) and CVS females were mated with nïve males and the litter size assessed. CVS females carried a significantly higher number of pups in the first parturition cycle (18.5 ± 1.6) as compared to naïve females (12.34 ± 2.35). No such effect was observed in the 2nd and 3rd parturition cycles (13 ± 1.18 and 11.14 ± 1.77 pups in the 2nd and 13.33 ± 0.88 and 12.5 ± 3.5 pups in the 3rd, respectively). The average litter size of 7 naïve and 8 CVS females is presented in each point. **C** Examination of the oviductal ampulae revealed that CVS females ovulated a significantly higher number of oocytes in their first estrous cycle (22.29 ± 1.97) as compared to naïve females (14.83 ± 1.51). The average ovulation of 8 naïve and 7 CVS females is presented in each point

At the end of the CVS protocol, the females were mated with males that did not undergo CVS (naïve males, Fig. 1A). As reported previously by us and others, no significant difference in the percentage of females with normal estrous cycle length and cyclicity was observed between naïve and females undergoing CVS [21, 24]. Surprisingly, CVS females exhibited a significantly higher litter size on their first parturition cycle following treatment (Fig. 1B). Pups weight and gender distribution were unaffected (supplementary Figures 3 A-B). No differences in litter size were found in later parturition cycles (Fig. 1B). In agreement with the increased litter size, CVS-exposed females ovulated a significantly higher number of oocytes in the first estrous cycle following treatment (Fig. 1C).

**Fig. 2:**
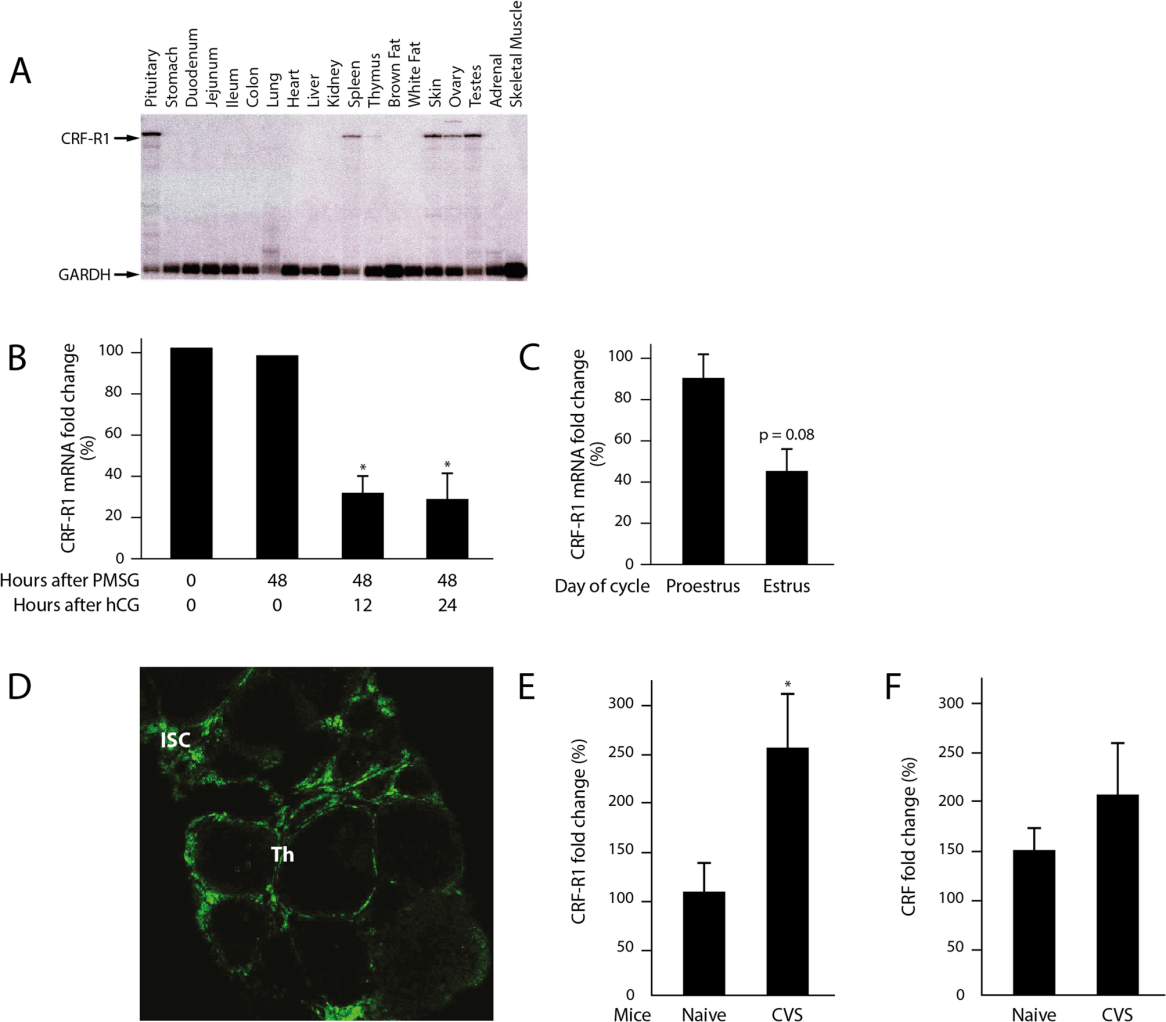
Spatio-temporal characterization of CRFR1 in the ovary. **A** CRFR1 expression in different tissues. A robust expression of CRFR1 was detected in the pituitary and in the gonads. **B** CRFR1 expression in ovaries of immature female mice, stimulated to ovulate by exogenous hormones administration (Superovulation, see Materials and Methods). CRFR1 levels are not affected by PMSG but sharply decrease after hCG administration. All time points were normalized to time zero of hCG (48 h after PMSG administration, 7 animals per each time point). **C** CRFR1 expression in ovaries of adult cycling mice. The ovarian CRFR1 expression is lower on the estrous as compared to the proestrous day. (proestrus and estrous 10 females each) (**D**) Localization of CRFR1 in the ovary. CRFR1 is expressed exclusively in the interstitial and theca cells of the follicles. It cannot be detected in the granulosa cells and oocytes. ISC-interstitial cells, Th- theca cells. **E** The ovaries of female mice subjected to CVS express a significantly higher level of CRFR1 mRNA at the first proestrous day after treatment as compared to naïve females (naïve 6 and CVS 10 females) (**F**) CRF expression in the ovaries of female mice subjected to CVS is elevated at the first proestrous day after treatment. (naïve 6 and CVS 10 females). In all experiments mean ± SEM is presented. *= *p* < 0.05

The expression of CRFR1 mRNA in the mouse pituitary was high, as expected (Fig. 2A). However, unexpectedly, an abundant expression of CRFR1 was also detected in the gonads of sexually mature female mice (Fig. 2A). We further found that the expression of CRF-R1 in the ovary is hormonally regulated. In the superovulation model, the high gonadal expression of CRFR1 in these mice was unaffected by pregnant mare serum gonadotropin (PMSG, an FSH–like hormone), administered to induce antral follicle development, but decreased sharply at 12 h after the following administration of human chorionic gonadotropin (hCG, an LH-like hormone), for induction of ovulation (Fig. 2B). In agreement with this, a high ovarian CRFR1 mRNA expression was also found in the ovaries of sexually mature mice at proestrus, when elevated FSH levels are found (Fig. 2C). This was followed by a decrease in CRF-R1 mRNA levels at estrus, the day of the cycle on which the LH-surge takes place (Fig. 2C).

To reveal the localization of CRF-R1 in the ovary, in the absence of a reliable antibody for immunostaining, we used the well characterized mouse model expressing GFP in a cellular distribution that largely mimics that of CRFR1 mRNA and is extensively colocalized with CRF-R1 [19]. Examining ovarian sections of sexually mature females of this model, we localized the ovarian expression of CRFR1 in mice exclusively the thecal and interstitial cells (Fig. 2Da-b). This result is similar to previous reports in rats and humans [14, 16].

Our analysis of the effect of CVS on ovarian CRFR1 expression revealed, on the proestrous-day, a significant elevation in its mRNA levels in the ovary of sexually mature ICR females, subjected to CVS as compared to naïve mice (Fig. 2E). These findings agree with a previous report that following stress, CRFR1 mRNA signal in the ovarian theca-interstitial layer is stronger [15]. We also observed a somewhat higher expression of CRF in those ovaries of CVS-exposed female (Fig. 2F). Taken together, these findings may point at an intra-ovarian environment, which influences the stress-induced transcription of the gonadal CRFR1.

**Fig. 3:**
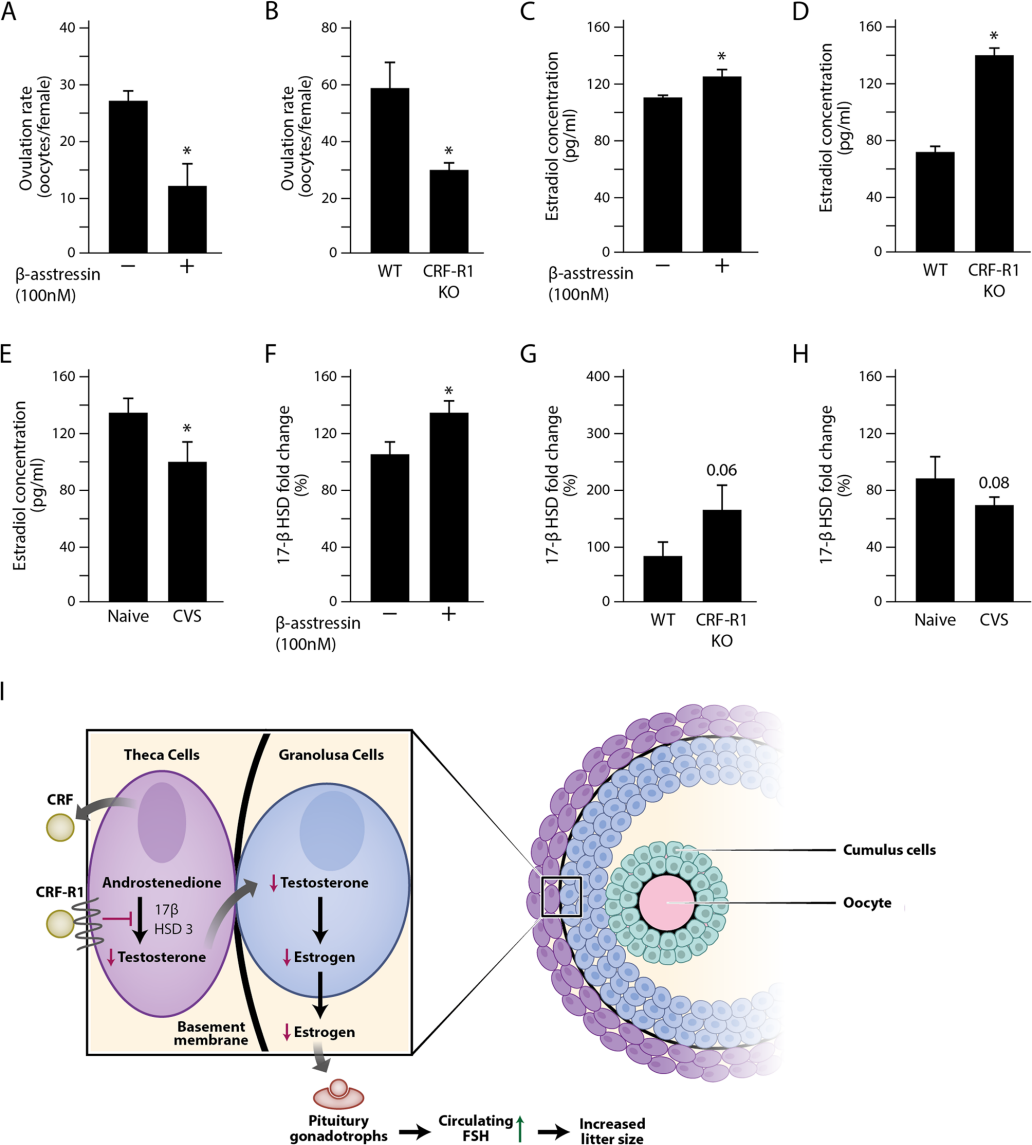
CRFR1 controls the levels of ovulation and that of serum estradiol (**A**) WT females, hormonally treated for the induction of ovulation and subjected to ovarian intrabursal injection of the CRF inhibitor β-asstressin, showed a significantly lower (11.86 ± 3.86) ovulation rate as compared to controls (26.63 ± 2.47, 8 non treated females and 7 females treated with β-asstressin) (**B**) Hormonally treated null females lacking the expression of CRFR1 exhibit a significantly lower number of ovulated oocytes (30.11 ± 2.28) as compared to WT (58 ± 8.61). (naïve and CRFR1 KO 8 females each) (**C**) Intrabursal injection of β-asstressin to WT animals, hormonally treated for induction of ovulation, significantly increases the serum levels of estradiol (β-asstressin treated and non-treated, 8 females each) (**D**) Hormonally treated CRFR1 KO females exhibit significantly higher serum estradiol concentrations as compare to WT. (WT and CRFR1 KO 6 females each) (**E**) WT females exposed to CVS contain a significantly lower serum estradiol levels. (naïve 6 and CVS 9 females) (**F**) mRNA levels of HSD17β3 in the ovary is significantly higher in hormonally treated females injected with β-asstressin. (β-asstressin and non-treated, 8 females each) (**G**) Ovaries of hormonally treated CRFR1 KO females express higher levels of HSD17β3 as compared to WT. (WT and CRFR1 KO 8 females each) (**H**) Ovaries of CVS-exposed females express lower levels of HSD17β3 mRNA (naïve 6 and CVS 10 females). In all experiments the mean ± SEM from at least three independent experiments is presented. *= *p* < 0.05 (**I**) A proposed model for the role of CRF and its ovarian receptor (see details under Discussion)

Involvement of the ovarian CRF in ovulation has been proposed by several previous studies [18, 25–27]. To investigate the local role of CRFR1 in mediating the CRF signal we injected β-asstressin, a nonselective CRF receptor antagonist into the ovarian bursa of superovulated animals. This treatment significantly reduced the ovulation rate in the treated animals as compared to controls (Fig. 3A). A similar, significant reduction in ovulation rate was observed in superovulated CRFR1 KO females (Fig. 3B). This result complements our findings that the CVS-induced increase in CRFR1 expression is associated with an elevated rate of ovulation (Figs. 1C and 2E, respectively).

These above findings, as well as other studies, also suggested a direct effect of CRF on the sex steroids output in ovarian cells [18, 25, 26, 28–31]. Supporting this idea in our experimental models, intraovarian β-asstressin injected animals as well as CRFR1 KO females, demonstrated that ablation of CRFR1 activity significantly elevated serum estradiol (Fig. 3C and D, respectively). Our CVS-exposed mice provide further substantiation for this notion; as opposed to the above models, these mice express high levels of CRFR1 (Fig. 2E), with a corresponding decrease in circulating estrogen (Fig. 3E). These findings agree with other reports of CRF suppressing estrogen production in vitro in mouse follicles [28], in rat and human granulosa-lutein cells [29, 30] as well as in human placenta trophoblast cells [32]. Moreover, our results demonstrate for the first time an in vivo effect of CRF receptors on ovarian steroidogenesis.

In the ovary, androgens produced in the theca are transferred to the granulosa cells, where they undergo aromatization, converting to estrogens. Our findings, showing that CRFR1 is localized in the theca cells (Fig. 2D), combined with our result that modifying CRFR1 activity affects estrogen levels (Fig. 3C-E), led us explore the expression of 17β-hydroxy steroid dehydrogenase type 3 (HSD17β3), a key enzyme in testosterone production in the theca cells. We detected higher expression levels of HSD17β3 mRNA in both, superovulated and β-asstressin-injected animals as well as in CRFR1 KO females compared to WT mice (Figs. 3F-G). We further found that ovaries of mice undergoing CVS and expressing high CRFR1 levels (Fig. 2E), exhibited reduced HSD17β3 (Fig. 3H). Either CRFR1 inhibition in vivo, by β-asstressin or its deletion in CRFR1 KO females, did not affect aromatase mRNA levels (supplementary Figures 4 A-B). Our data suggests, therefore, that CRFR1 effect on serum estradiol is not mediated by neither aromatase expression nor its activity, as suggested previously [29, 30], but rather represents the negative control of this CRFR on HSD17β3 expression and the resulting testosterone production in the theca cells. These findings are in line with a previous study showing that Urocortin1, another CRF family member, inhibited Leydig cell function and testosterone production [33]. This study also demonstrated that Urocortin1 activity is mediated by its binding to the CRFR1 receptor [33]. Testicular Leydig cells are derived from the same embryonic origin as the ovarian theca cells. Our results thus suggest a CRF/CRFR1 autocrine/paracrine negative effect on HSD17β3 expression in the theca cells manifested, in turn, by lowering serum estradiol.

Based on our results we propose the following model. a CRF family ligand secreted from either the ovary or another source, binds to CRFR1 localized on the theca cells. In a mechanism, yet to be elucidated, this binding leads to downregulation of HSD17β3 followed by a decline in testosterone availability and a subsequent reduction in ovarian estrogen production (Fig. 3I). According to this suggested mechanism the elevation in CRFR1 expression, upon exposure to CVS, leads to a reduction in HSD17β3, which in turn, brings about a decreased estrogen production. The decline in circulating estrogen, removes its negative feedback on FSH release from the pituitary (Fig. 3I). As it is the level of FSH that determines the size of ovulation [34], under these conditions, the number of ovulated oocytes is increased. Supporting this model, previous studies have demonstrated that high corticosteroid levels stimulate the release of FSH [3].

**Fig. 4:**
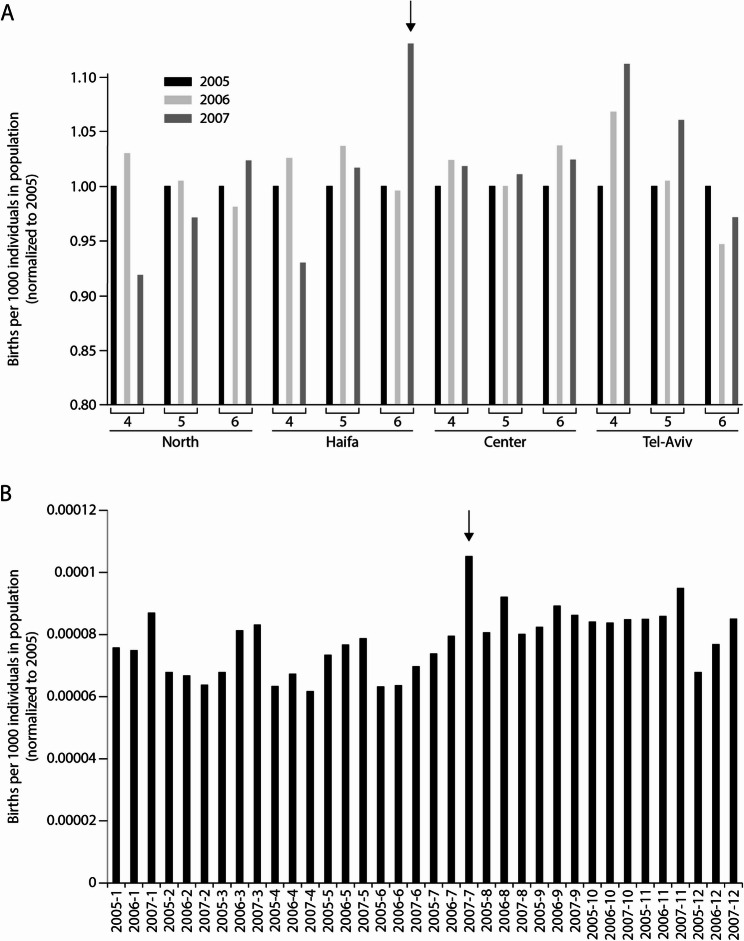
“Baby-boom” in Israel after the 2nd Lebanon war. **A **An elevation in birth rate is seen in Haifa, but not in Tel Aviv, 9 months after the second Lebanon war. **B** A particular increase in multiple-births rate, indicating a higher incidence of ovulation of more than a single oocyte, is observed in Israel, 9 months after this stressor event (see arrow)

Several examples along our recent history display that a dramatic increase in birth rate follows stressor events. Such phenomena, known as “Baby Boom”, were reported following the 2nd World War as well as throughout the collapse of Czechoslovakia [35, 36]. Although well-known and robust, its physiological and biological background has not been studied yet. We claim herein that increase in birth rate following stressor events represents, at least in part, a higher incidence of multiple ovulations. A most recent demonstration of such phenomenon is registered following the second Lebanon war, taking place in our region during August to October 2006. Our analysis of the Israel Central Bureau of Statistics data reveals a peak in birth rate in Haifa, which was the target of a massive missiles attack, but not in Tel Aviv (Fig. 4A). More strikingly, supporting the idea of an increase in the size of ovulation in individual females, 9–10 months after the war, we identified a peak in the number of multiple births (twins and triplets) (Fig. 4B). In that respect, examination of the Israel Central Bureau of Statistics data for the number of multiple births, soon after the Covid19 pandemic, which is not yet available, would be of enormous interest.

## Discussion

Unlike the common knowledge that stress is accompanied by disruption of reproductive functions, we show herein a beneficial effect of CVS on ovulation and the subsequent litter size. We further demonstrate that this gonadal response is mediated by upregulated local expression of CRFR1 in ovarian cells, followed by downregulation of estrogen production. Our suggested mechanism for the effect of CRF/CRFR1 in the mouse ovary may suggest that the “Baby Boom” phenomenon does not only represent a social reaction to stress, but, in addition, a physiological element to this phenomenon. It is possible that, like mice, women temporarily exposed to long unexpected stressors (such as war, missile attacks etc.), upregulate the expression of their ovarian CRFR1. This elevation would lead to a reduced estrogen production, followed by a higher FSH secretion. This, in turn, could lead to an elevated ovulatory response, and the subsequent increase in the incidence of multiple-birth pregnancies in a mechanism described in Fig. 3I. The suggested role of CRF in the ovary as presented here, may also contribute to our understanding of polycystic ovarian syndrome (PCOS). It has been reported that in PCOS patients the ovarian theca cells express and secret lower CRF levels [14]. According to our findings, this may account for the irregular menstrual cycle, anovulation, hirsutism and depression [37] which are associated with the high androgen levels produced by their ovaries [14, 38]. A previous study further suggested that rape-pregnancy rate are higher than pregnancies from consensual sex [39]. This finding is supported by our findings that CVS elevates fertility. In our study CVS led to reduced estrogen levels, while other study demonstrated that estrogen can blunt the stress response in women [40]. Taken together, it can be suggested that the reduction of estrogen observed by us might be necessary for the CVS-induced ovulation and fertility rate. Finally, our finding that CVS elevated ovulation rate is supported by previous studies showing that acute stress may stimulate the release of LH from the pituitary leading to induction of ovulation in rats, rhesus monkeys and women [41]. In addition, one of our previous studies demonstrated that exposure to CVS leads to an increased response to stress by modulating gene expression in the PVN, the main region of CRF production [21]. Interestingly, this study further showed that females with history of exposure to CVS had higher response and greater sensitivity in their GABAergic neurons. GABA has been shown previuously to stimulate CRF secretion and activity [42]. Therefore, it might be that history of exposure to CVS affects later events in the organism life, such as response to stress and reproduction, by increasing the sensitivity of different cells and organs, like the PVN or the ovary.

## Conclusion

Our results propose a novel mechanism for the local influence of CRF in the ovary that can contribute substantially to the understanding of the effects of stress on both, normal and pathological ovarian functions. These results may possibly lead to the development of novel means to alleviate the clinical fertility.

## Supplementary Information

Supplementary Material 1.

Supplementary Material 2.

## Data Availability

No datasets were generated or analysed during the current study.

## Abbreviations

CRF: Corticotropin releasing factor
CRFR1: CRF receptor type 1
CRFR1 KO: CRFF1 knock out
CVS: Chronic variable stress
FSH: Follicle stimulating hormone
hCG: Human chorionic gonadotropin
HSD17b3: 17β-hydroxy steroid dehydrogenase type 3
PMSG: Pregnant mare serum gonadotropin
PCOS: Polycystic ovarian syndrome
PVN: Paraventricular nucleus
WT: Wild type
