# Supplementary figures and images for: Mild chronic stress promotes female fertility via the ovarian CRF receptor
Source: Cell Commun Signal. 2025 Aug 14;23:372. doi: 10.1186/s12964-025-02371-0 (PMC12351781; doi:10.1186/s12964-025-02371-0)

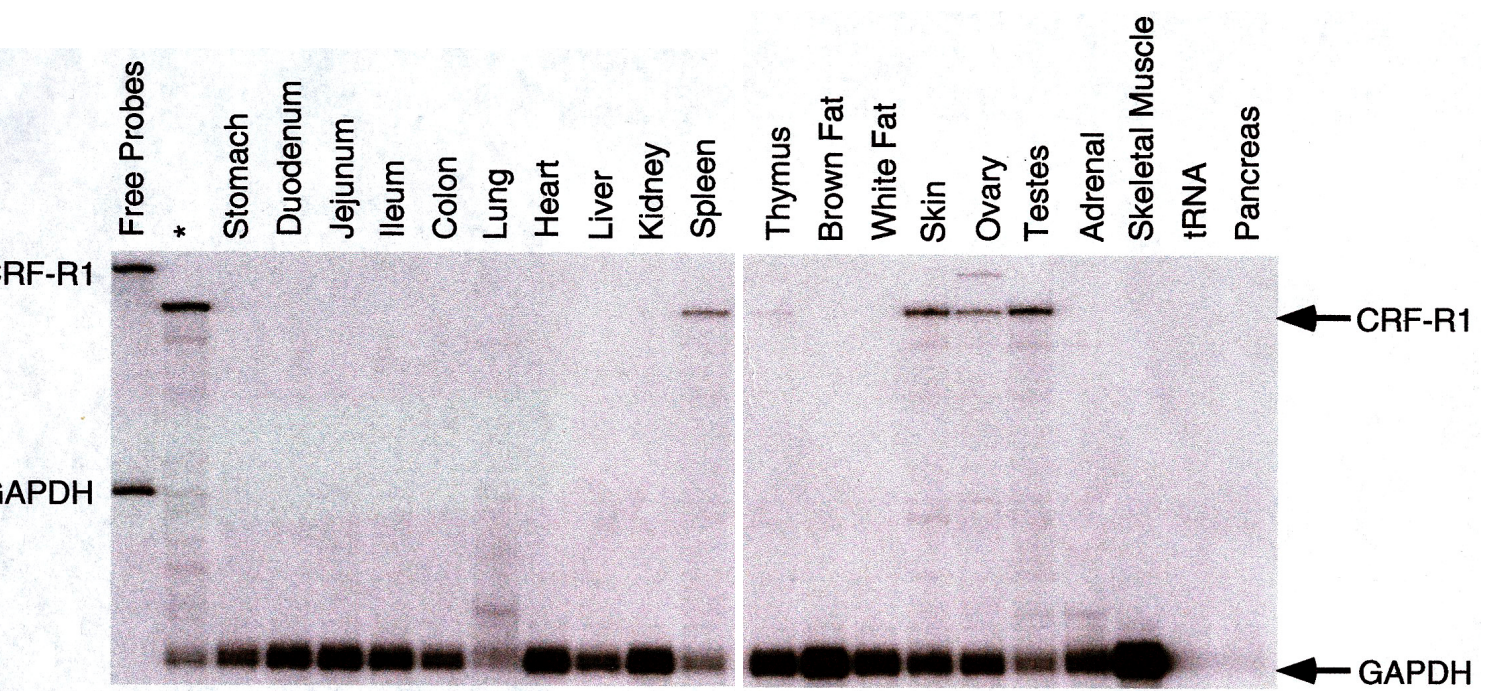

Supplement: Supplementary file 1 — Supplementary Material 1. [file 12964_2025_2371_MOESM1_ESM.pdf]
