# Supplementary material for: Mild chronic stress promotes female fertility via the ovarian CRF receptor
Source: Cell Commun Signal. 2025 Aug 14;23:372. doi: 10.1186/s12964-025-02371-0 (PMC12351781; doi:10.1186/s12964-025-02371-0)
Supplement: Supplementary file 2 — Supplementary Material 2. [file 12964_2025_2371_MOESM2_ESM.pdf]

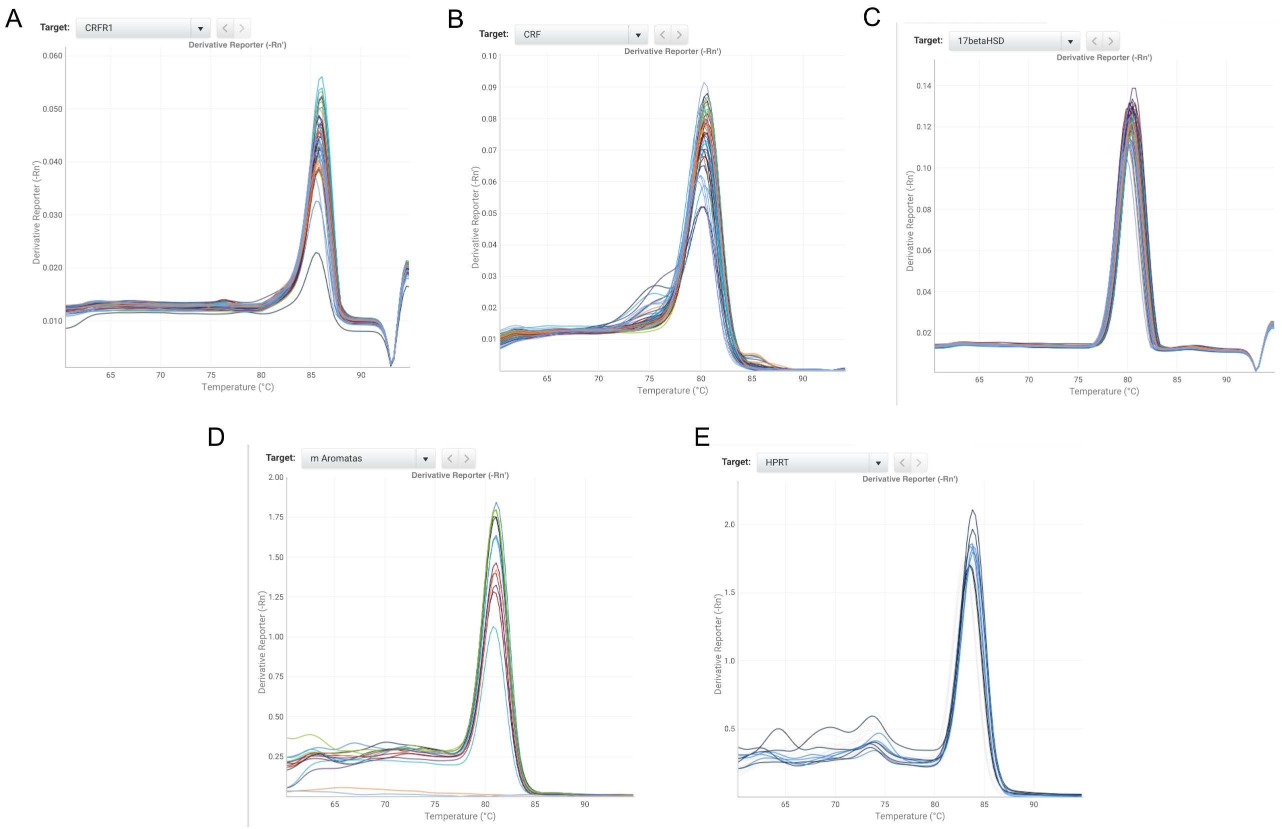

Supplementary figure S1. Melt curve of all genes and primers used in this study. A) CRF-R1, B) CRF, C) 17ß-HSD, D) AROMATASE, E) HPRT

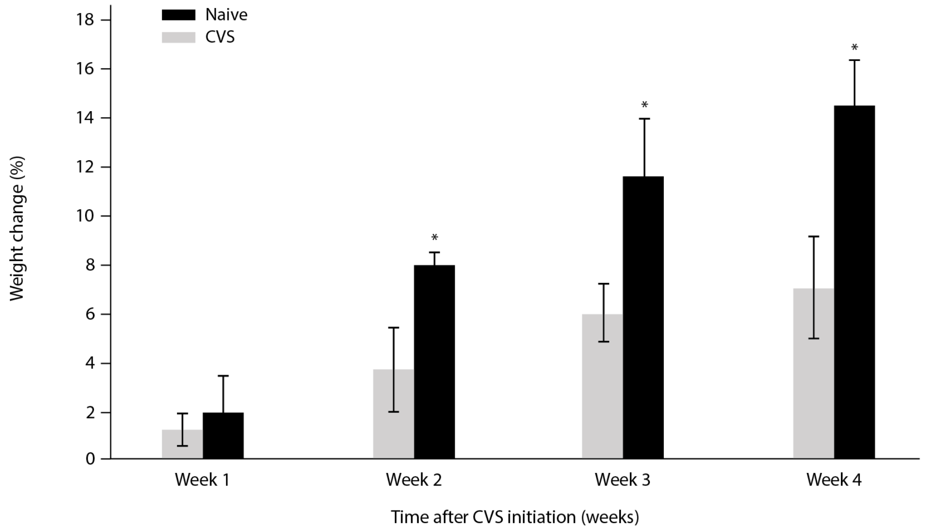

Supplementary figure S2. A lower weight gain is observed in CVS through the stress period as compared to naive mice. Percentage of weight change compared to the weight at the starting point is presented. (21 naïve females and 25 CVS exposed females). Values show the means ± SEM. *= p<0.05.

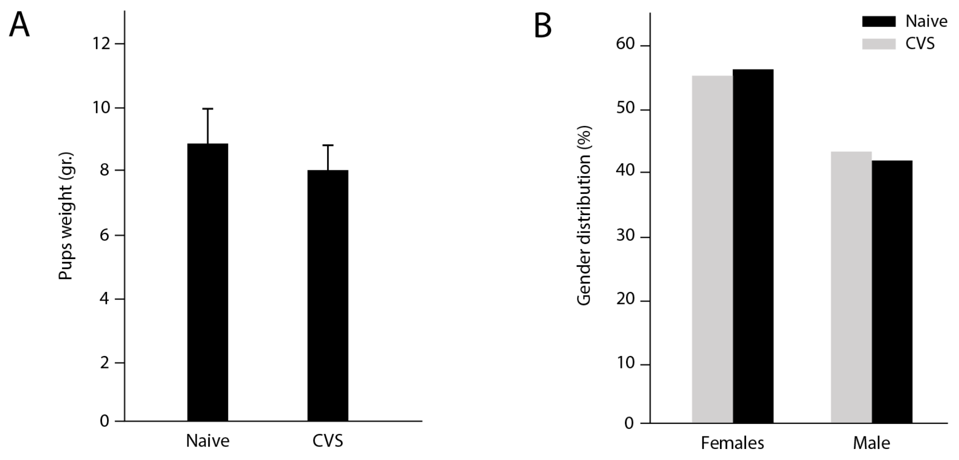

Supplementary figure S3. The pups of control and CVS females were weighed and their gender examined at weaning. No differences in weight of pups of naïve vs. CVS females were found (A). No variations in gender distribution in the litters of naive and CVS females were found (B). (CVS, n=72; control, n=40).

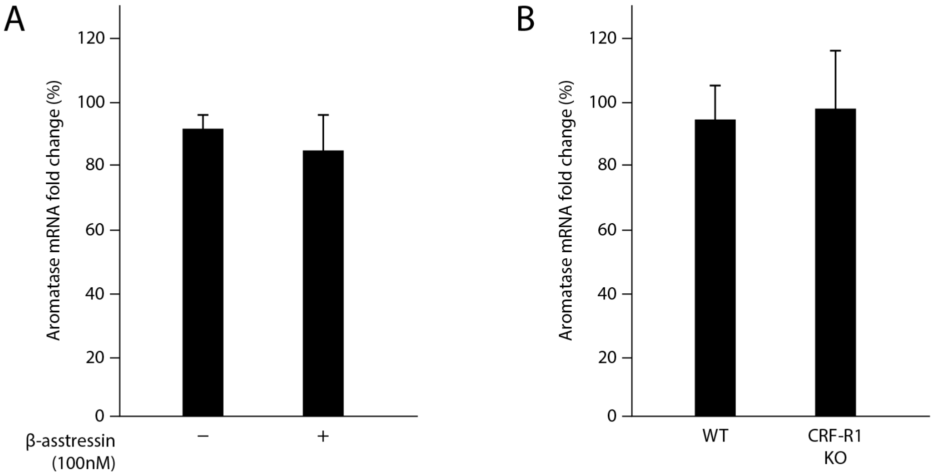

Supplementary figure S4. No difference in aromatase expression was found in the ovaries of WT and β-asstressin treated and untreated mice (A, 8 females non treated and 8 females treated with β-asstressin), as well as ovaries of WT and CRFR1 KO females (B, n=8 for each WT and CRF-R1 KO groups).
